# Person-identifying brainprints are stably embedded in EEG mindprints

**DOI:** 10.1038/s41598-022-21384-0

**Published:** 2022-10-11

**Authors:** Yao-Yuan Yang, Angel Hsing-Chi Hwang, Chien-Te Wu, Tsung-Ren Huang

**Affiliations:** 1 Department of Computer Science and Engineering, University of California San Diego, La Jolla, USA; 2 Department of Communication, Cornell University, Ithaca, USA; 3 International Research Center for Neurointelligence (WPI-IRCN), The University of Tokyo Institutes for Advanced Study (UTIAS), The University of Tokyo, Tokyo, Japan; 4 Department of Psychology, National Taiwan University, Taipei City, Taiwan; 5 Neurobiology and Cognitive Science Center, National Taiwan University, Taipei City, Taiwan; 6 Institute of Applied Mathematical Sciences, National Taiwan University, Taipei City, Taiwan

**Keywords:** Cognitive neuroscience, Computational neuroscience

## Abstract

Electroencephalography (EEG) signals measured under fixed conditions have been exploited as biometric identifiers. However, what contributes to the uniqueness of one's brain signals remains unclear. In the present research, we conducted a multi-task and multi-week EEG study with ten pairs of monozygotic (MZ) twins to examine the nature and components of person-identifiable brain signals. Through machine-learning analyses, we uncovered a person-identifying EEG component that served as "base signals" shared across tasks and weeks. Such task invariance and temporal stability suggest that these person-identifying EEG characteristics are more of structural brainprints than functional mindprints. Moreover, while these base signals were more similar within than between MZ twins, it was still possible to distinguish twin siblings, particularly using EEG signals coming primarily from late rather than early developed areas in the brain. Besides theoretical clarifications, the discovery of the EEG base signals has practical implications for privacy protection and the application of brain-computer interfaces.

What do our brains reveal about us as individuals? While most neuroimaging studies investigate brain connectivity and activity common across individuals, these neural patterns can reveal individual uniqueness. Earlier studies found that brain structures, activation patterns during cognitive tasks, and functional connectivity could account for individual differences in personality^1,2^, intelligence^3–5^, emotional processing^6–8^, and psychopathological conditions^9,10^. In fact, these brain characteristics were sufficiently distinct to be exploited as biometric identifiers^11,12^.

The unique brain characteristics used for personal identification can be measured using structural or functional neuroimaging methods. For example, magnetic resonance imaging (MRI) studies have found brain sizes, shapes, and volumes to be informative of person identity^12^; electroencephalography (EEG) and functional MRI (fMRI) studies have found brain activities under certain conditions (e.g., during a resting, task-performing, or emotional state) to be distinct for each individual^13–16^. Although the terms "brain fingerprints" or "brainprints" have been used loosely to refer to either structural or functional brain characteristics by some of the personal identification studies^12,13^, we use "brainprints" and "mindprints" to describe unique signatures of brain structures and mental processing, respectively.

Notably, while brainprints or brain structural signatures are primarily determined by genes and measured by structural neuroimaging, brain structures and brainprints can be shaped by experiences via neuroplasticity and reflected in functional neuroimaging^17,18^. For example, as early as the 1930s, scientists had observed nearly identical resting-state EEG patterns within Monozygotic (MZ) twin pairs^19^. By contrast, this extent of similarity was neither observed between dizygotic (DZ) twin pairs nor among non-related people. Importantly, despite that EEG profiles appear to be predominantly determined by genetic factors^20,21^, they are also found to be influenced by environmental factors during brain development^20–22^. Therefore, one's EEG signals are a complex manifestation of both mindprints and brainprints resulting from both nature and nurture (Fig. 1).

**Figure 1:**
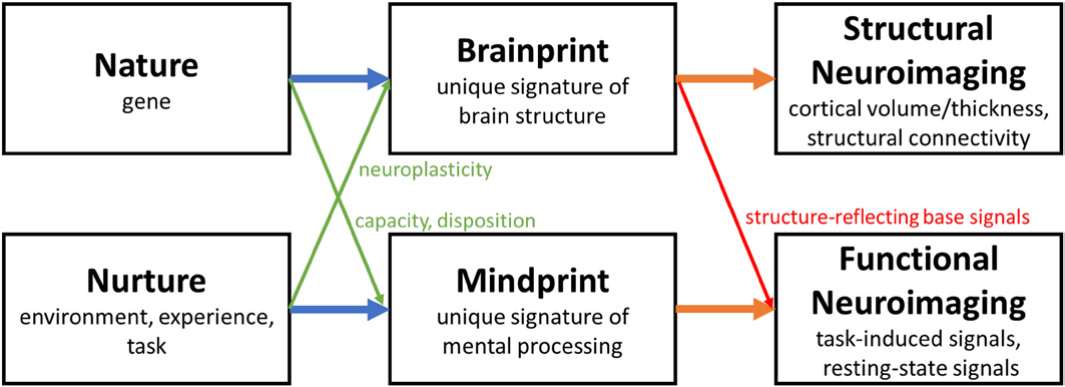
Conceptual illustration of brainprint versus mindprint. While structure-reflecting brainprints are often measured by structural neuroimaging, the current study observes brainprints in the signals of functional neuroimaging. Moreover, both nature and nurture can shape brainprints and mindprints.

Given the complex composition of one's EEG signals, it remains unclear whether person-identifying EEG characteristics are more of mindprints or brainprints and result from genes or environments. Clarifying the nature of person-identifying EEG components helps not only to advance our understanding of individual differences or inter-subject variability in functional brain imaging but also to potentially develop better brain-computer interfaces that can generalize and personalize well at the same time. Therefore, the present study takes a close examination of the composition of person-identifying EEG signals to address the aforementioned questions.

While person-identifying EEG characteristics, being functional signals, appear to reflect mindprints, we hypothesize the opposite—person-identifying EEG characteristics may reflect one's brainprints embedded in mindprints, serving as "base signals" shared across different mental processes. Such a hypothesis is derived from an often-neglected fact that resting is an inhomogeneous task condition during which one can freely jump from one thought to another as if they are undergoing different cognitive or emotional tasks. Thus, the findings of person-identifying resting-state EEG signals hint toward a common person-identifying EEG component shared across different task conditions.

To test our hypothesis, we applied machine learning methods on EEG spectral features to search for the existence of person-identifying "base signals" across various tasks (see Methods for details). In other words, unlike previous studies that restricted their search of brainprints/mindprints under specific tasks or the resting condition^16^, the present study intermixed EEG samples from different task conditions as the data for model training and testing. Specifically, we collected multi-week EEG data from ten monozygotic (MZ) twin pairs (N = 20) and carried out systematic machine-learning investigations to examine the stability and origin of these "base signals" across tasks, weeks, and twins.

## Methods

### Ethics and participants

All the study protocols were reviewed and approved by the Institutional Review Board at National Taiwan University Hospital (protocol #: 201604024RIND), and all experimental procedures followed relevant guidelines and regulations for protecting human participants. 10 pairs of MZ twins (gender: 14 females; age: M = 20.5, S.D. = 1.7) were enrolled in the present study. All 20 participants signed an informed consent form before the experiment. Each participant received a compensation of $2,400 NTD in cash for four recording sessions. Each recording session was one week apart from another, and the whole investigation spanned over one month for each participant. In total, our dataset contains 80 EEG recording sessions (20 twin participants * 4 weekly sessions).

### Procedures and tasks

The experiment consists of four EEG recording sessions, which are referred to as Week 1 ~ Week 4 in this article. Each of the two successive sessions was separated by a week for us to study the temporal stability of brainprints and mindprints. Prior to participation, the participants were informed detailed instructions for each task condition, followed by practices to confirm their complete understanding of the instructions.

Each session contained four instructed-task blocks and one free-task block. Each instructed block included ten 65-s, randomly ordered task conditions: 1. resting with eyes closed, 2. resting with eyes opened, 3. repeated opening and closing of left palm, 4. repeated opening and closing of right palm, 5. motor imagery of repeated opening and closing of left palm, 6. motor imagery of repeated opening and closing of right palm, 7. repeated opening and closing of both palms, 8. repeated plantarflexion and dorsiflexion of both feet, 9. motor imagery of repeated opening and closing of both palms, 10. motor imagery of repeated plantarflexion and dorsiflexion of both feet. During the free-task block, the participants could do anything they liked while remaining seated for 5 min (e.g., using their smartphone to read or play games).

### Data collection and preprocessing

EEG signals were recorded with a 32-channel Quick-Cap connected to a SynAmps 2 amplifier (Compumedics NeuroScan, USA). Two electrodes positioned at the left and right mastoid were used as references for off-line analysis (the right mastoid was the online reference), and one electrode at the forehead was used as a ground. Vertical eye movements (vEOG) were recorded with two electrodes placed above and below the left eye, and horizontal eye movements (hEOG) were recorded with two electrodes placed on the outer canthus of the left and right eye. The impedance was kept below 5 kΩ for all electrodes by applying Quick-Gel. The EEGs were sampled at a rate of 500 Hz with an online bandpass filter of 0.01 to 100 Hz.

None of the 30 nonreference EEG electrodes was labeled as a bad channel and excluded from further analysis. In each recording block, no channel had more than 15% excessively fluctuating data points with 5 standard deviations from the mean averaged across channels at a given time. In other words, no channel was distinct from other channels throughout each recording block to serve as a person-identifying artifact stably. Note, however, that our very first participant's fourth block of the first session was not recorded because of technical issues.

Next, we used the MNE-Python toolbox v0.24.1^23^ to remove eye-related artifacts from our EEG data. Specifically, the EEG data from all recording sessions (i.e., different tasks and weeks) were first concatenated for a given participant as the inputs to an independent component analysis (ICA). With an adaptive outlier threshold defaulted at z-score = 3.0 for Pearson correlations, the MNE-Python toolbox then auto-identified components that were extremely correlated with eye-related activities in electrooculography (i.e., EOG from HEO and VEO channels). After removing these eye movement artifacts, we reconstructed the eye-unrelated EEG signals with the remaining independent components for personal identification.

After the preprocessing, we verified the existence of the Berger effect and mu suppression in our EEG data as sanity checks of data quality^24,25^. The Berger effect is a well-established EEG phenomenon that refers to the increase in the alpha-band power over the occipital/parietal areas when individuals close their eyes compared to when they open their eyes. The mu suppression refers to the decrease in the mu-alpha power over the sensorimotor cortex when individuals perform actual movements or motor imagery. As shown in the supplementary information, both the Berger effect (Figures S1 & S2) and the mu suppression (Figure S3) were observed. Therefore, all subsequent analyses were conducted on quality EEG data rather than artifactual noise.

### Person sampling

With 10 pairs of twin participants, we often sampled 10 out of 20 participants to examine person identification with or without twin siblings in our computational experiments. To identify non-twin individuals, we sampled one person from each of the 10 twin pairs. To identify twin siblings, we sampled 5 out of 10 twin pairs. Because the identification accuracy was affected by person sampling, throughout the main texts and supplementary information, we always reported the mean accuracy and standard error computed from 20 repetitions of 10-person sampling. By contrast, we reported only one accuracy for 20-person identification because there was no sampling involved.

### Analysis pipeline

The protocol for training and evaluating the classifiers for EEG-based personal identification is detailed below. First, we standardized the time series and converted them into spectrograms. Next, we extracted the power spectral density of each 2-s segment into one feature vector. These feature vectors were then used as person-representing data samples to train a classifier. During model testing, a trained classifier first made a raw prediction on each unseen sample. Then, we combined 15 raw predictions from temporally neighboring samples into one final prediction via majority voting. Throughout the present study, we used the percentage of correct final predictions to evaluate the degree to which a machine classifier could accurately identify the participants. For convenience, we refer to this percentage as *identification accuracy* or *recognition accuracy*.

### Feature extraction

Because there are individual differences in EEG oscillations^26,27^ that characterize brain functions^28–30^, we constructed spectral feature vectors from raw EEG data as person-representing samples for training and testing a classifier. During this feature extraction process, we used the whole dataset or a subset of it to train and test a machine classifier depending on the purpose of each computational experiment. Each subset could be selected based on the weeks when data were collected, tasks participants performed, individuals being twin or non-twin, or EEG channels.

The feature extraction process consisted of several steps. First, we z-scored the data for each channel. Next, these standardized signals from each channel were converted into a spectrogram comprising a time series of power spectral density (PSD), each of which was calculated from a 2-s segment of 1,000 samples in the time domain with an overlap of 500 samples (i.e., 1 s) between two neighboring segments. Based on our preliminary results now shown in this article, we selected only the most identity-predictive parts of each PSD—theta (4 ~ 8 Hz), alpha (8 ~ 12 Hz), and beta (12 ~ 29 Hz) bands—to be spectral features. Finally, such spectral features from 30 EEG channels were concatenated to form a feature vector to characterize each 2-s EEG segment except for the cross-channel experiments (Figs. 6 and 7). In total, a 65-s task would yield 64 such task-representing feature vectors for model training or testing.

### Data splits and classifier training

**Figure 2:**
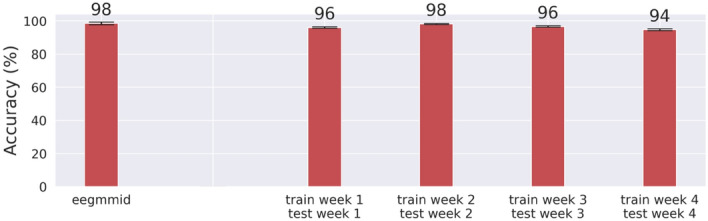
Single-day personal identification. The means of 10-person identification accuracies and their standard errors resulted from the single-day EEGMMID data (the leftmost bar) and our multi-day EEG data (the four bars on the right).

We used the extracted feature vectors as data samples to train and test machine classifiers for personal identification. Except for the single-day personal identification (Fig. 2), all our computational experiments used data from Week 1–3 for modeling tuning/training and data from Week 4 for model testing.

Specifically, we performed classification using the one-versus-all L2-regularized logistic regression from the scikit-learn package^31^. Its regularization strength parameter, C, was determined by a grid search for optimizing its mean classification accuracy in a tenfold cross-validation procedure on the data from Week 1–3, with 64 samples of the same task shuffled as a group. Other hyperparameters that controlled the machine learning process—such as tolerance for stopping criteria and class weights—were kept as the default values set by the scikit-learn package. Finally, one classifier with these determined hyperparameters was trained on all the data from Week 1–3 and subsequently tested on all the data from Week 4. We adopted this classification model to compute identification accuracy for all the analyses except those otherwise stated.

We chose logistic regression as the classifier because of its simplicity. It required less computational power to carry out all our computational experiments and allowed us to easily visualize the person-identifying "base signals" (Fig. 7). While other advanced machine learners, such as support vector classifiers or deep-learning neural networks, may also be used to further boost person identification accuracy at the cost of computational efficiency, the simple logistic regression was sufficient to provide the existence proofs of the EEG base signals.

### Voting process for model predictions

As one 2-s sample might be insufficient for accurate personal identification, we combined the predictions of temporally consecutive samples into a final prediction via a majority voting process. Specifically, the 64 testing samples from the same task originally produced 64 raw predictions, which were then aggregated by a sliding window of 15 samples to produce 50 final predictions. The final predictions significantly improved the accuracies of raw predictions. For example, the accuracy of personal identification using eyes-closed resting data went up from 84.81 to 94.87% correct for twins and from 91.57 to 98.98% correct for non-twins. Therefore, it was the accuracy of final rather than raw predictions being reported throughout this article.

## Results

Various sets of results are presented in different sections. We start with results of single-day personal identification using data collected by us and others. Next, we report supporting evidence for the existence of person-identifying EEG components that are task-independent, temporally invariant, and spatially distributed across all the EEG channels. Finally, we address the origins of these "base signals" by examining the spectral and spatial differences of these person-identifying signals.

### Single-day personal identification

To verify the quality of our EEG data and ensure the generality of our analysis pipeline, we carried out the same analysis on our multi-day data and the single-day data from EEGMMID^32^ to see if we could obtain comparable personal identification accuracies from these two datasets. The publicly available EEGMMID contains complete EEG data from 105 research participants performing an array of simple motor or motor imagery tasks, which are similar to those used in the present study. For a fair comparison, we randomly sampled 10 out of the 105 participants from the EEGMMID data and 10 non-twin individuals from our data (i.e., one individual from each of our ten twin pairs) for personal identification. Moreover, one machine classifier was trained on the first two out of three blocks of EEGMMID data corresponding to the task of "*opening and closing both fists or both feet*," and another machine classifier was trained on the first two blocks of our data corresponding to the tasks of "*moving both fists*" and "*moving both feet*" in the current study. These two classifiers were then tested on their own held-out data for performance comparison. Figure 2 summarizes the personal identification results, which confirm the good quality of our data and the generalizability of our analysis pipeline.

### Cross-day personal identification

**Figure 3:**
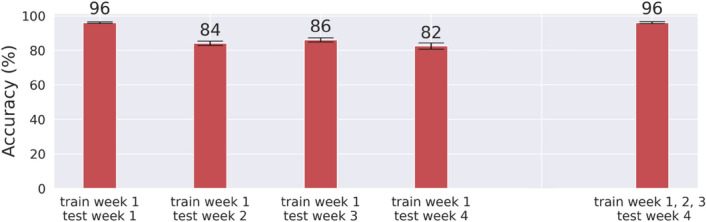
Cross-week personal identification. The means of 10-person identification accuracies and their standard errors resulted from single-day (the four bars on the left) and multi-day EEG data (the rightmost bar).

It was unclear whether the person-identifying features found in the single-day EEG signals were as temporally stable as fingerprints to serve as biometric brainprints/mindprints. To address this question, we trained a machine-learning classifier using data from the first day and tested it on data from another day, up to four weeks apart. This was to examine whether the day-specific EEG features picked by the classifier remained robust to identify participants on data collected on different days/weeks. For a performance comparison with Fig. 2, we also randomly sampled one person out of each twin pair and analyzed the EEG data from the periods of "*moving both fists*" and "*moving both feet*." The mean testing accuracies and their standard errors are shown in Fig. 3.

While all identification accuracies remained significantly higher than chance (10% accurate) in Fig. 3, the identification accuracy degraded as training and testing data were further apart in time, suggesting a slow drifting of such person-identifying brain activities. To mitigate this performance degradation problem, we hereafter trained our machine learners using data from multiple weeks to capture the time-independent components of person-identifying EEG signals. Specifically, we used data collected from Week 1–3 for model training and those from Week 4 for model testing throughout the rest of our reported analyses.

### Cross-task personal identification

**Figure 4:**
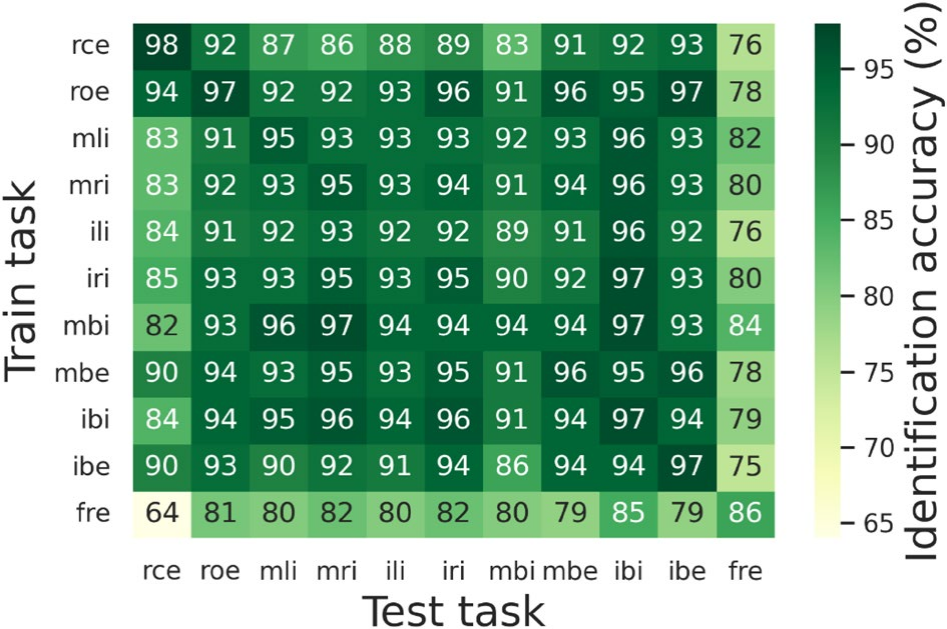
Cross-task personal identification. Each cell in the matrix indicates the mean 10-person identification accuracy of a particular train-test combination. rce = resting with closed eyes, roe = resting with opened eyes, mli = moving left fist, mri = moving right fist, ili = imagining moving left fist, iri = imagining moving right fist, mbi = moving both fists, mbe = moving both feet, ibi = imagining moving both fists, mbe = imagining both feet, fre = free task.

Having examined the temporal stability of the person-identifying features in EEG, we then took a further step to investigate whether such features found in one task from Week 1–3 can be used to identify participants in another task from Week 4. Following the same procedure for cross-day personal identification, we randomly sampled one participant out of each twin pair to train and test our classification model. Figure 4 summarizes the mean testing accuracy of each train-test combination of tasks.

Overall, the identification accuracies increased marginally when the models were tested on the same task (i.e., diagonal numbers in Fig. 4) than tested on different tasks (i.e., off-diagonal numbers in Fig. 4). Remarkably, the identification accuracies were all much higher than chance (10% correct) when the models were tested on the "*free task,*" during which participants could do whatever they liked while remaining seated, such as reading and playing games on their smartphones. Taken together, these results suggest the existence of a person-identifiable EEG component commonly shared by different tasks.

### Cross-twin personal identification

We further examined the model performance of identifying twin versus non-twin individuals using the EEG data during the eye-closed resting task. To allow for a fair performance comparison between models that distinguished twins versus non-twins, we equalized the number of individuals to be identified in the two experimental conditions. Specifically, we sampled five out of the ten twin pairs for the twin condition (N = 10) and one participant out of each twin pair for the non-twin condition (N = 10). The mean model testing accuracies and their standard errors were 98.98 ± 0.21% and 94.87 ± 0.89% for the non-twin and twin identification conditions, respectively.

**Figure 5:**
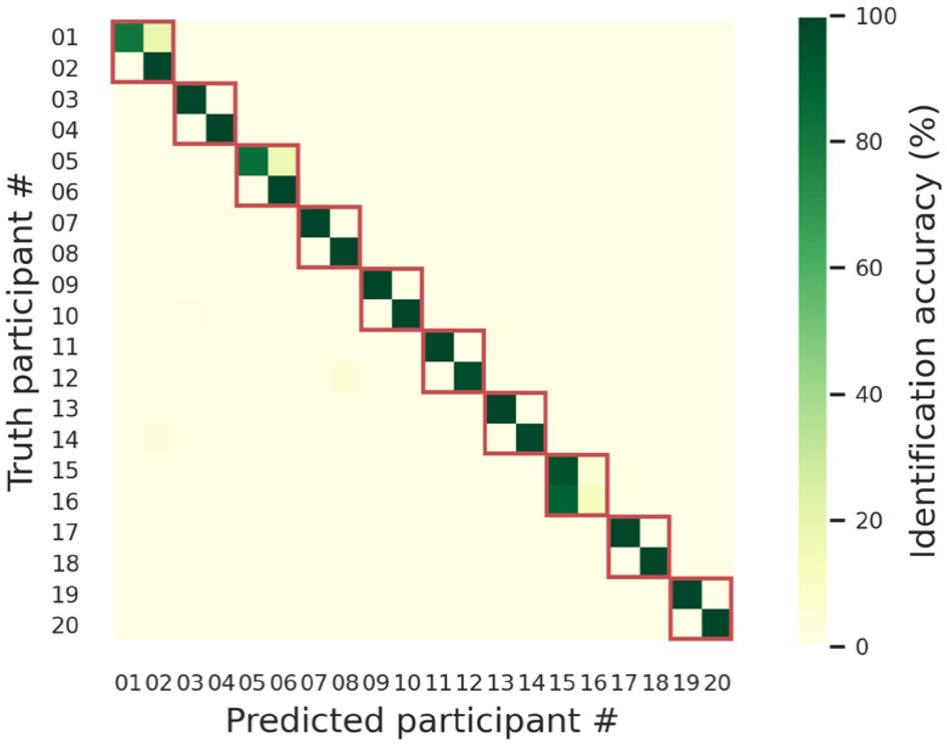
Cross-twin personal identification. Each cell in the confusion matrix indicates the percentage of the model predicting a particular participant (horizontal axis) given the EEG features of a particular participant (vertical axis). Each row was normalized by the total number of instances in that row to factor out the sampling-induced, inequal number of total samples across rows. Note that two consecutive participant IDs correspond to a twin pair (e.g., participants #1 and #2 are one twin pair, and participants #3 and #4 are another pair). The red boxes highlight the confusion submatrix within twin pairs.

To understand why the model poorly performed when identifying twin participants, we carried out another computational experiment using the whole dataset (N = 20) and examined the confusion matrix of this 20-person identification experiment. As shown by the confusion matrix in Fig. 5, although the machine classifiers were more confused by within-twin than between-twin individuals (i.e., dark-green diagonal elements and light-green non-diagonal elements in Fig. 5), the overall accuracy of classifying data from Week 4 was still as high as 93.33%. In other words, the person-identifying components of EEG signals were similar but not totally identical in twins, possibly influenced by both nature and nurture.

### Cross-channel personal identification

While the person-identifying components in EEG were found to be temporally stable and shared across tasks, it remained unclear whether such EEG characteristics were spatially local or global features. Previously, the feature vector representing each person comprised signals from different EEG channels, and the machine classifiers could pick up channel-wise features for personal identification. Therefore, instead of concatenating data from different channels into a feature vector, we constructed 30 channel-wise feature vectors as training samples for each time window so that a machine classifier could learn to discriminate individuals using features shared across 30 EEG channels. In this way, the number of training samples would, however, become 30 times larger than before. For computational efficiency, the sliding time windows were changed from 50- to 0%-overlapping between the two consecutive windows, which yielded a sample size only 15 times larger than before.

**Figure 6:**
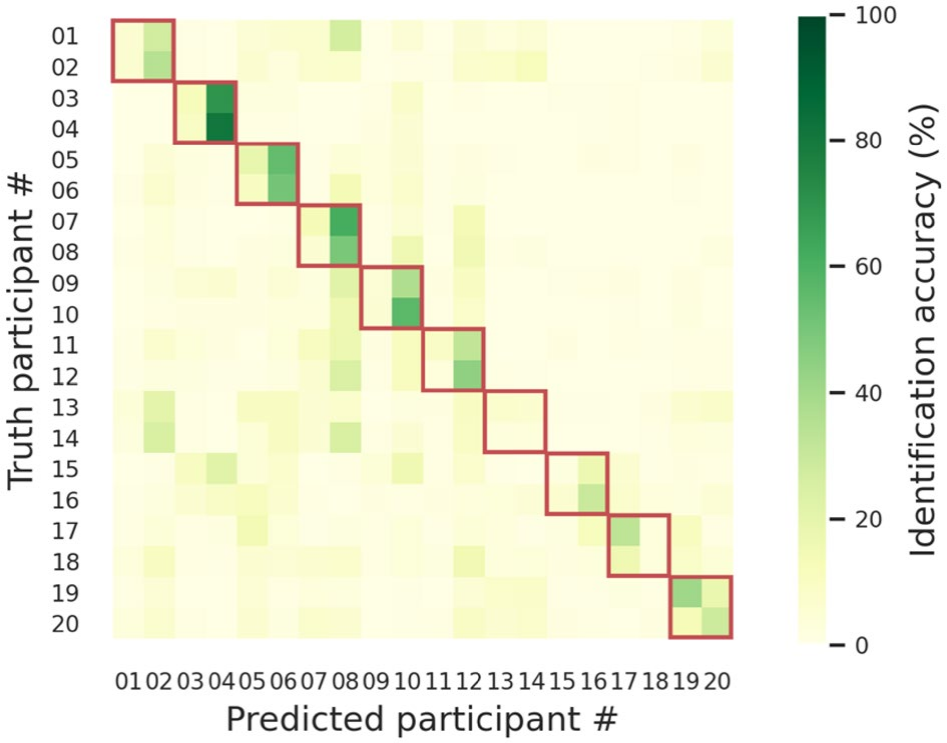
Cross-channel personal identification. Each cell in the confusion matrix indicates the percentage of the model predicting a particular participant (horizontal axis) given the EEG features of a particular participant (vertical axis). Each row was normalized by the total number of instances in that row to factor out the sampling-induced, inequal number of total samples across rows. Note that two consecutive participant IDs correspond to a twin pair (e.g., participants #1 and #2 are one twin pair, and participants #3 and #4 are another pair). The red boxes highlight the confusion submatrix within twin pairs.

The channel-wise feature vectors extracted from four representative tasks (i.e., *resting with eyes closed*, *resting with eyes open*, *moving both fists*, & *imagining moving both fists*) were then used as training and testing samples for a machine classifier to identify ten pairs of twins (N = 20). Figure 6 presents the confusion matrix of the experiment. The model attained an overall identification accuracy of 26.80% for the training data from Week 1–3 and 26.22% for the testing data from Week 4. Such identification accuracies were not as high as the results from channel-mixed feature vectors but were still considerably higher than chance (i.e., 5% correct). These results prove the existence of person-identifying "base signals" shared across all EEG channels.

### Spectral differences in base signals

In this section, we attempted to scrutinize the nature of the person-identifying "base signals." The relatively simple machine classifiers we used in the previous section—logistic regression—allowed us to easily visualize the person-identifying features shared across days, tasks, and EEG channels. Specifically, because we employed a one-versus-all classification approach, each logistic regression model would learn to weight features (i.e., power spectral densities) as pieces of evidence for predicting whether a given EEG feature vector is extracted from a specific person. Therefore, although the weight vectors are not person-identifying "base signals" per se, they can be seen as the cartoon version of the "base signals," which emphasizes the diagnostic features of a person while deemphasizing features commonly shared by others.

**Figure 7:**
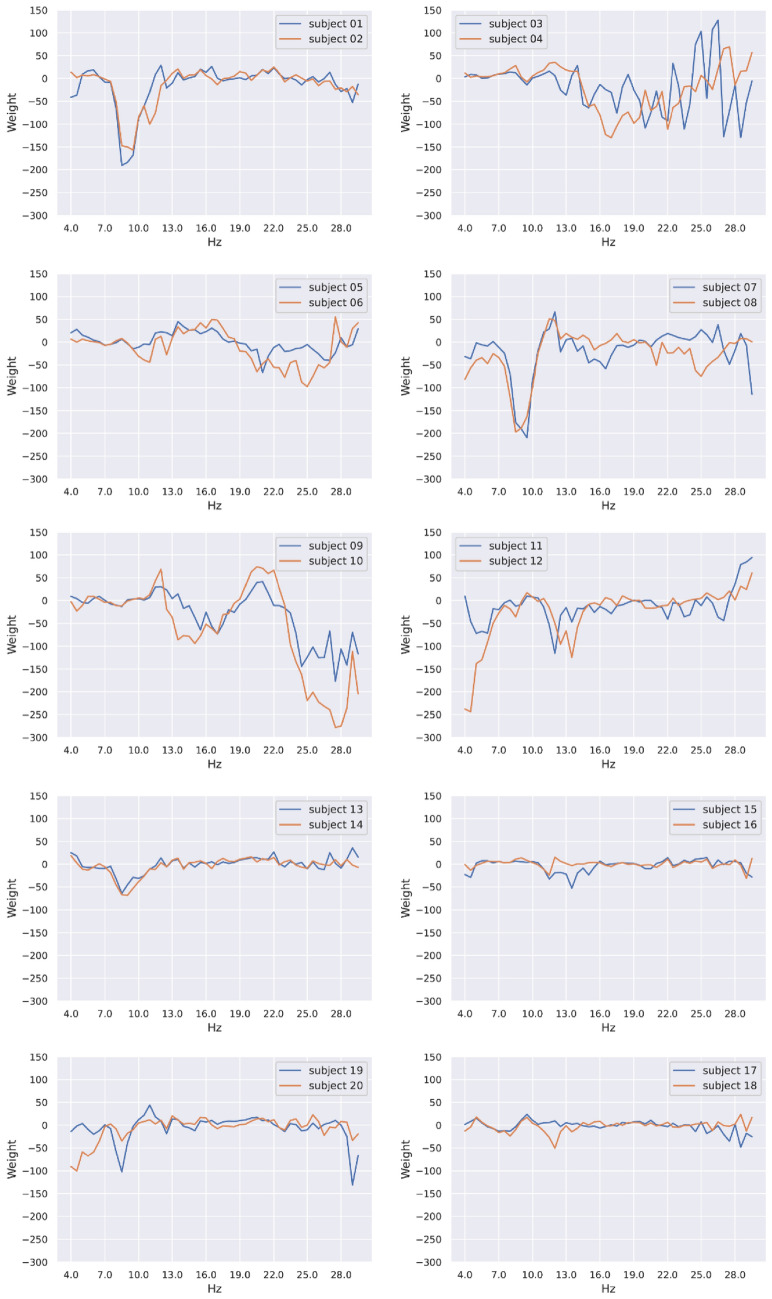
Spectral differences in base signals. Each panel visualizes the frequency-based weight vectors our machine classifier used to characterize the two individuals of each MZ twin pair.

We visualized the values of the weight vector associated with each person-detecting classifier in Fig. 7. In the figure, positive or negative weights indicate the corresponding features being treated, respectively, as positive or negative evidence of a person. The magnitude of each weight indicates how much a classifier relies on the corresponding feature to make judgments, highlighting the diagnostic frequencies of each individual. Overall, the results in Fig. 7 echoed the results in Figs. 5 & 6—patterns of weight vectors were similar within twins but quite distinct between twins, and hence it was more confusable when discriminating between twin siblings and non-twin individuals.

### Spatial differences in base signals

While genes might set the main tone for the EEG base signals, later development of the brain might introduce minor changes in the base signals that our machine learners exploited to differentiate twin siblings. To investigate such developmental contributions to base signals, we further examined the differentiability of person-identifying EEG signals from the channels corresponding to early and late matured areas in the brain^33,34^. Specifically, instead of using all the EEG channels for constructing a feature vector, we carried out personal identification using regional EEG signals only from the prefrontal, parietal, temporal, or occipital channels. We expected that signals from later developed, more experience-shaped brain areas (e.g., prefrontal cortices^34^) would be overall more person-identifying than those from early developed, less experience-shaped brain areas (e.g., occipital cortex).

Table 1 summarizes the personal identification results based on regional EEG signals of four representative tasks (two resting and two motor-related). In the table, the mean accuracies of identifying data from Week 4 and their standard errors were calculated from 20 repetitions of 10-person sampling. Indeed, the personal identifications based on the frontal EEG channels were higher in accuracy than those based on other channels, regardless of whether the identification targets were twins or not. In other words, the EEG channels that primarily originated from later developed prefrontal areas provided more person-identifying signals than did early developed brain areas. This result suggests that nurture, in succession to nature, further shapes person-identifying EEG signals and makes highly similar EEG signals within twins more differentiable.

**Table 1:** Spatial differences in base signals.

	Task	Frontal	Parietal	Temporal	Central	Occipital
**Non-twin**	rce	**99.10 ± 0.15%**	92.31 ± 0.57%	96.10 ± 0.51%	93.30 ± 0.49%	80.61 ± 1.44%
	roe	**96.17 ± 0.52%**	86.25 ± 0.90%	87.88 ± 1.27%	88.03 ± 0.99%	65.82 ± 1.09%
	mbi	**92.05 ± 1.08%**	78.00 ± 1.10%	80.07 ± 1.57%	80.54 ± 0.81%	51.42 ± 1.43%
	ibi	**92.88 ± 1.10%**	87.55 ± 0.82%	87.18 ± 1.19%	85.87 ± 0.81%	70.11 ± 1.75%
**Twin**	rce	**96.84 ± 0.61%**	81.25 ± 1.38%	83.89 ± 1.58%	83.81 ± 0.77%	63.97 ± 1.10%
	roe	**95.76 ± 0.58%**	78.19 ± 1.24%	79.99 ± 1.40%	78.68 ± 1.11%	58.74 ± 1.88%
	mbi	**92.85 ± 0.64%**	65.64 ± 1.37%	67.17 ± 1.73%	67.39 ± 1.52%	42.47 ± 1.69%
	ibi	**93.25 ± 1.17%**	76.61 ± 1.42%	77.03 ± 1.11%	79.79 ± 1.10%	63.99 ± 1.60%

Each cell in the table shows the mean accuracy and its standard error of 10-person identification using EEG data from a particular task (row) and brain region (column). rce = resting with closed eyes, roe = resting with opened eyes, mbi = moving both fists, ibi = imagining moving both fists.

For each task, the best region-based identification results are highlighted in bold.

## Discussion

The current study discovers person-identifying EEG "base signals," which can serve as a basis for personal identification in previous EEG studies. Remarkably, these EEG base signals are temporally stable for a month (Fig. 3), contextually independent of tasks (Fig. 4), and spatially shared across channels (Fig. 6). Moreover, these base signals are more similar within than between MZ twins (Fig. 7), resulting in lower identification accuracies within each twin pair (Figs. 5 and 6). However, it is still possible to distinguish between twin siblings, particularly using EEG signals coming primarily from late rather than early developed areas in the brain (Table 1).

Importantly, these results are not primarily driven by non-brain components in the EEG signals. First of all, the Berger and mu suppression effects observed in our preprocessed data (see the *Data Collection & Preprocessing* section under Methods) ensured that we worked with quality EEG data rather than artifactual noise. Secondly, physiological signals such as heart activity (< 240 bpm or 4 Hz) or muscle activity (> 20 Hz) could not contribute to the high accuracy of person identification based on spectral features ranging from 4 to 20 Hz (see the *Person Identification without High-beta Band* section in the Supplementary Information). Thirdly, eye-related EEG signals could not fully account for the high identification accuracy, either (see the *EOG-based Person Identification* section in the Supplementary Information). Finally, a person's head shape could give rise to a unique profile of inter-electrode distances/correlations for that person. However, such a covariance structure among electrodes, if any, was unavailable to our machine classifier, which could successfully identify individuals based on spectral features shared across rather than concatenated from all EEG channels (see the “Cross-channel personal identification” section under “Results”). Therefore, we also ruled out differences in head shape as the sole driver of our results.

While EEG records functional signals, the person-identifying EEG base signals are not function-reflecting mindprints. First of all, the person-identifying base signals are found to be shared across function-specific tasks. Therefore, they are unlikely to subserve any particular brain function. Furthermore, the successful extraction of the person-identifying base signals from voluntary, function-nonspecific tasks corroborates the function-independency of the base signals. Finally, the higher personal identification accuracies achieved by resting-state than task-based signals (as shown in Fig. 4 and Table 1) imply that active brain functioning masks rather than contributes to the person-identifying base signals.

The nature of task-independent, person-identifying base signals in EEG may seem perplexing but can be understood as structure-reflecting brainprints. Just as resting-state functional connectivity reflects structural connectivity in MRI^17,18^, functional EEG signals can also reflect structural characteristics in the brain in addition to task-relevant characteristics. In the present study, the most person-identifying signals are the resting-state EEG signals and thus more like brainprints than mindprints. Also, similar to the case where person-identifying, content-independent acoustic fingerprints reflect the structures of vocal cords, the person-identifying, task-independent EEG base signals may reflect brainprints embedded in mindprints.

The structure-reflecting brainprints manifested in EEG can be shaped by both nature and nurture. For example, while genes guide the initial development of the brain structures, life experiences and neuroplasticity continuously change structural connectivity in the brain^18^. In the present study, while the EEG base signals, in terms of their spectral uniqueness, are much more similar between MZ twin siblings than between non-twin individuals, we cannot conclude that genes outweigh life experiences in the contribution to these person-identifying EEG signals. This is because each pair of our twin participants was raised in the same household, and hence the high similarity of the EEG base signals between the MZ twin siblings can be contributed by both genetic and environmental factors. It still awaits future investigations to clarify the relative importance of nature and nurture in shaping the person-identifying EEG base signals.

## Conclusion

The discovery of the person-identifying EEG base signals has implications both for the science and application of EEG. Scientifically, removing such person-specific components of EEG can help remove undesirable inter-subject variability in event-related potential (ERP) studies and de-identify EEG data before data sharing. The person-specific components of EEG, once isolated, can also be used to study individual differences or pathological characteristics. For applications, removing the person-specific components of EEG during the development of an EEG-based brain-computer interface (BCI) can help improve the BCI's initial generalizability across individuals. Conversely, the person-specific components of EEG can be later identified and added to the aforementioned BCI system for personalization.

In conclusion, the existence of the person identifying EEG base signals is an important finding, which has advanced our understanding of EEG signals as a dynamic composition of both brainprints and mindprints. We hope that the present study has paved the way for future studies to use such base signals to their full potential.

## Supplementary Information

Supplementary Information.
