# Supplementary material for: Person-identifying brainprints are stably embedded in EEG mindprints
Source: Sci Rep. 2022 Oct 11;12:17031. doi: 10.1038/s41598-022-21384-0 (PMC9553892; doi:10.1038/s41598-022-21384-0)
Supplement: Supplementary file 1 — Supplementary Information. [file 41598_2022_21384_MOESM1_ESM.docx]

Supplementary Information of

**Person-identifying brainprints are stably embedded in EEG mindprints**

Yao-Yuan Yang, Angel Hsing-Chi Hwang, Chien-Te Wu, Tsung-Ren Huang

**EEG Data Quality**

We carried out two sanity checks to verify the quality of our EEG data. The first one is the Berger effect, which refers to the increase in power of the alpha-band oscillation (8~12 Hz) over the occipital/parietal areas when individuals close their eyes compared to when they open their eyes. The second one is mu suppression, which refers to the decrease in the power of the mu-alpha oscillation (8~12 Hz) over the sensorimotor cortex when individuals perform actual movements or motor imagery. Both the Berger and the mu suppression effects are well-established phenomena in the EEG literature.

To show the Berger effect, we averaged the ratio of the spectral power of the resting-with–eyes-closed (EC) task to that of the resting-with-eyes-open (EO) task over all our participants for each recorded session. The ratio was indeed significantly larger in the alpha band (8~12Hz) than in the other bands, showing an increase in power of the alpha band during the EC task compared to the EO task (Figure S1).


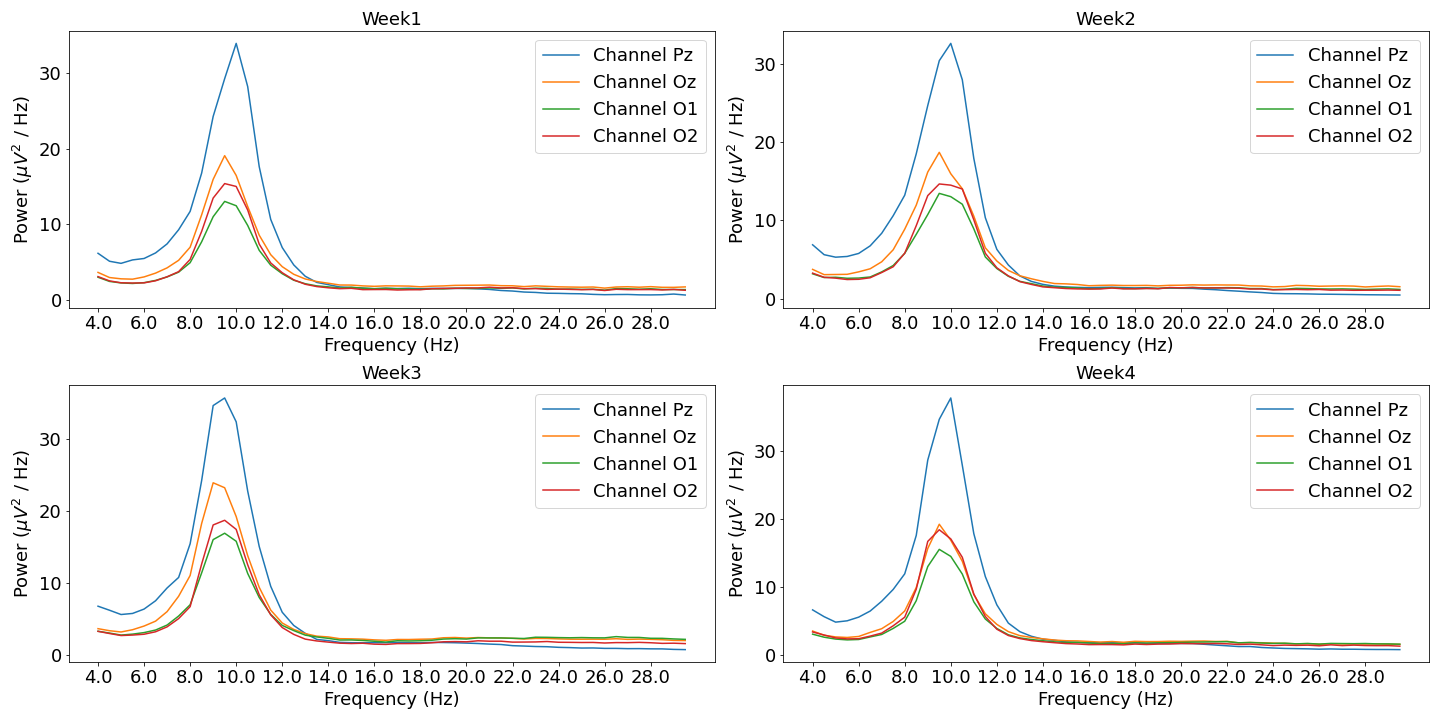


**Figure S1. The Berger effect at the group level.** For all the Pz, Oz, O1, & O2 electrodes in each recorded session/day, the averaged ratio of the spectral power of the resting-with-eyes-closed task to that of the resting-with-eyes-open task was significantly higher in the alpha bands (8~12Hz) than the other bands.

Moreover, the Berger effect was observed not only at the group level but also at the individual level. As summarized in Figure S2, the ratios of the alpha-band power of the EC task to that of the EO task were higher than one for all the participants. In other words, the alpha-band power increased during the EC task compared to the EO task for all our participants.


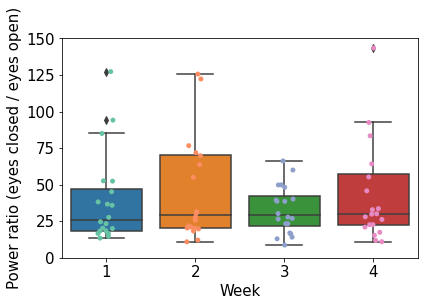


**Figure S2. The Berger effect at the individual level.** Each data point represents one participant in this box plot. The alpha-band power ratios were averaged across the Pz, Oz, O1, & O2 electrodes for each recorded session/day.

To show the mu suppression effect, we calculated the mu-band power of the "moving left fist" (mli) task in contrast to that of the "moving right fist" (mri) task, as well as the alpha-band power of the "imagining moving left fist" (ili) task in contrast to the "imagining moving right fist" (iri) task. Specifically, we first averaged 65 alpha-band powers calculated from 1-second, non-overlapping sliding windows across each 65-second task. Then, the differences in the power of the mli-mri contrast and the lil-iri contrast were further averaged across 20 participants into topographic maps in Figure S3. For both the contrasts, we indeed observed right-lateralized mu suppressions, namely reductions in the mu-alpha (8~12Hz) power of the EEG sensors near the right sensorimotor cortex.

| 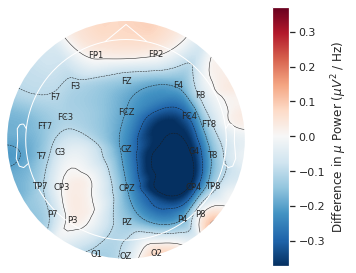 | 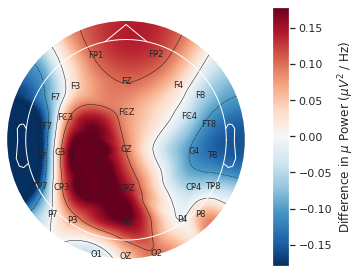 |
| --- | --- |
| (a) mli – mri | (b) ili - iri |

**Figure S3. The mu suppression effect.** (a) The topographic map of the mli-mri contrast. (b) The topographic map of the ili-iri contrast. **mli**=moving left fist, **mri**=moving right fist, **ili**=imagining moving left fist, **iri**=imagining moving right fist.

Both the Berger and mu suppression effects shown in our preprocessed data provide evidence that we were working with quality EEG signals rather than artifactual noise. More importantly, because artifactual noise can hardly be stable across channels, tasks, and weeks, the stable person-identifying EEG component found in the present study more likely captures person-identifying brainprints rather than artifactual noise.

**Empirical Chance Level**

While the theoretical chance level is 100/N% correct for N-person identification, we computationally established the empirical chance level of our machine-learning analysis pipeline. This was to examine whether our analysis pipeline would optimistically report better-than-chance accuracy even when there was no relationship between features and personal identity. Here we used the 20-person identification in Figure 5 as an example. After we randomly permuted the person labels in data, the mean identification accuracy and stand error dropped to 5.26% correct for training data and 4.71% correct for testing data. We further used the10-person identification of the "resting with closed eyes" (rce) condition in Table 1 as another example. After we randomly permuted the person labels in data, the mean testing accuracy and stand error became 10.03±0.06% correct for recognizing non-twins and 9.97±0.06% correct for recognizing twins. As evidenced by these results, the empirical chance level of our machine-learning pipeline was not optimistically biased.

**Identifying Individuals in EEGMMID**

To verify the generality of our analysis pipeline, we also carried out our analysis on the single-day EEG motor movement/imagery dataset (EEGMMID). Although EEGMMID contained EEG recordings of 109 research participants, the recorded signals of four participants (ID: 88, 92, 100, & 106) were incomplete and thus excluded from further analysis. In the end, 105 participants from EEGMMID were subjected to our personal identification pipeline, and the identification accuracy was 85.27% correct for the training data from Week 1–3 and 75.61% for the testing data from Week 4. Note, however, that the comparison of identification accuracy between the EEGMMID and our dataset in Figure 2 was based on 10-person sampling on each data set (see main texts for details).

**Person Identification without High-beta Band**

Muscle activity has a broad frequency distribution, starting from 20Hz. This frequency distribution overlaps with the high-beta band (20~29Hz) used as the spectral features for our machine classifier (see the *Feature Extraction* section under Methods). It is thus possible that person-identifying muscle activity rather than brain activity provided a basis for personal identification.


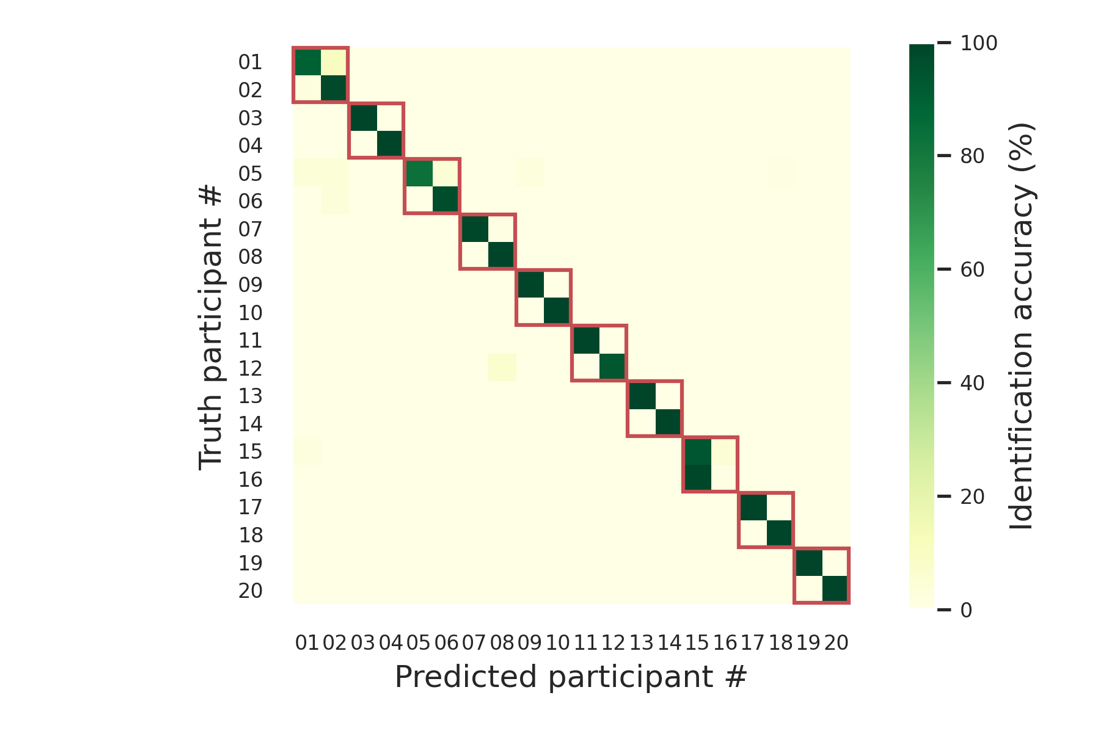


**Figure S4.** **Cross-twin personal identification without the high-beta band as features.** Each cell in the confusion matrix indicates the percentage of the model predicting a particular participant (horizontal axis) given the EEG features of a particular participant (vertical axis). Each row was normalized by the total number of instances in that row to factor out the sampling-induced, inequal number of total samples across rows. Note that two consecutive participant IDs correspond to a twin pair (e.g., participants #1 and #2 are one twin pair, and participants #3 and #4 are another pair). The red boxes highlight the confusion submatrix within twin pairs.

To test such a possibility, we used the 20-person identification in Figure 5 as an example and extracted spectral features only from 4~20Hz instead of the original range of 4~29Hz. After such a feature replacement, the overall accuracy of classifying data from Week 4 dropped slightly from 93.33% to 92.70% (Figure S4). In other words, the high identification accuracy we observed was not primarily driven by high-beta features or any muscle activities with a frequency higher than 20 Hz.

**EOG-based Personal Identification**

Although we have utilized ICA to remove eye-related components in our EEG signals (see the *Data Collection & Preprocessing* section under Methods), undetected eye-related activity could still survive in the signals to provide information about a person's identity. Therefore, it would be informative to examine how well the eye channels (vEOG and hEGO) alone could discriminate between participants. Here we used the 20-person identification in Figure 5 as an example and extracted spectral features only from the two eye channels (VEO & HEO) instead of the original thirty channels (see the *Feature Extraction* section under Methods). After such a feature replacement, the overall accuracy of classifying data from Week 4 dropped from 93.33% to 45.03%. In other words, the high identification accuracy we observed was not primarily driven by intact eye-related signals, let alone residual eye-related signals in our preprocessed data.
